# Differences in clinical aspects of human cystic echinococcosis caused by *Echinococcus granulosus sensu stricto* and the G6 genotype in Neuquén, Argentina

**DOI:** 10.1017/S0031182023000264

**Published:** 2023-06

**Authors:** María Florencia Debiaggi, Cristian A. Alvarez Rojas, Lorena Evelina Lazzarini, Daniel Calfunao, Paola Titanti, Liliana Calanni, Marisa Iacono, Silvia Viviana Soriano, Peter Deplazes, Nora Beatriz Pierangeli

**Affiliations:** 1 Cátedra de Microbiología y Parasitología, Facultad de Ciencias Médicas, Universidad Nacional del Comahue, Neuquén, Argentina; 2 Escuela de Medicina Veterinaria, Facultad de Agronomía e Ingeniería Forestal, Facultad de Ciencias Biológicas y Facultad de Medicina, Pontificia Universidad Católica de Chile, Santiago, Chile; 3 Servicio de Infectologia, Hospital Castro Rendon, Neuquen, Argentina; 4 Institute of Parasitology, Vetsuisse Faculty, University of Zürich, Zürich, Switzerland

**Keywords:** Clinical aspects, *cox1*, *Echinococcus granulosus sensu stricto*, *Echinococcus granulosus*, genotype G6, genotypes, human cystic echinococcosis

## Abstract

Most human cystic echinococcosis (CE) cases worldwide are attributed to *Echinococcus granulosus sensu stricto* (*s.s*), followed by the G6 and G7 genotypes. While *E. granulosus s.s.* has a cosmopolitan distribution, the G6 genotype is restricted to areas where camels and goats are present. Goats are the primary livestock in the Neuquén province in Argentina where the G6 genotype has been reported to be responsible for a significant percentage of CE human cysts genotyped. In the present study, we genotyped 124 *Echinococcus* cysts infecting 90 CE-confirmed patients. *Echinococcus granulosus s.s.* was identified in 51 patients (56.7%) with 81 cysts and the G6 genotype in 39 patients (43.3%) harbouring 43 cysts. Most CE cases ≤18 years were male suggesting pastoral work could be a risk factor for the infection. *Echinococcus granulosus s.s.* was significantly found more frequently in the liver (32/51 patients) and the G6 genotype in the lungs and extrahepatic localizations (27/39). The patients infected with *E. granulosus s.s*., presented up to 6 cysts while patients infected with G6 presented a maximum of 2. The diameter of lung cysts attributed to *E. granulosus s.s.* was significantly larger compared to lung cysts from G6. Following the WHO ultrasound classification of liver cysts, we observed inactive cysts in 55.6% of G6 cysts and only 15.3% of *E. granulosus s.s* cysts. In conclusion, we provide evidence of differences in clinical aspects of CE caused by *E. granulosus s.s.* and the G6 genotype of *E*. *granulosus s*.*l*. complex infecting humans.

## Graphical abstract

**Figure:**
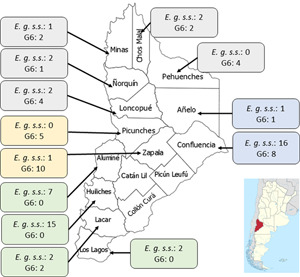

## Introduction

Cystic echinococcosis (CE) is a severe zoonosis distributed worldwide caused by species of the *Echinococcus granulosus sensu lato* (*s*.*l*.) complex (Deplazes *et al*., 2017). The current taxonomy of *E. granulosus s.l.* establishes strong delimitations between *Echinococcus granulosus sensu stricto* (*s*.*s.*) (which includes the genotypes G1/G3 and related variants), *Echinococcus equinus* (G4), *Echinococcus ortleppi* (G5) and the group comprising the G6/7/8/10 genotypes for which the nomenclature remains controversial (Lymbery *et al*., 2015; Nakao *et al*., 2015). Furthermore, the G6 and the G7 genotypes show clear differences in intermediate host specificity; G6 is commonly associated with camels and goats and G7 with pigs. Additionally, molecular data comparing sequences of the full length of the *cox1* and *nad* genes (Addy *et al*., 2017) and the complete mitochondrial genome (Laurimäe *et al*., 2018) confirmed the differentiation of both genotypes.

*Echinococcus granulosus s.s.* is accountable for the majority of human CE cysts genotyped worldwide (88.4%) followed by the G6 genotype (7.3%) (Alvarez Rojas *et al*., 2014). However, in some countries/geographic areas, the G6 genotype is the most commonly found variant of the parasite in humans including Egypt (Abdel Aaty *et al*., 2012; Alam-Eldin *et al*., 2015), Mauritania (Bardonnet *et al*., 2002, 2003; Bart *et al*., 2004; Maillard *et al*., 2007, 2009) and Sudan (Omer *et al*., 2010). The G6 genotype is also present, but is not the most commonly found variant of *E*. *granulosus*, in other countries/geographic areas including Iran (Shahnazi *et al*., 2011; Sadjjadi *et al*., 2013); Russia (Konyaev *et al*., 2012); Mongolia (Jabbar *et al*., 2011) and the Turkana region in Kenya (Casulli *et al*., 2010). In South America, the G6 genotype has been identified in a single human cyst in Chile (Manterola *et al*., 2008) and in 2 humans from Peru (Santivañez *et al*., 2008; Moro *et al*., 2009). In the Neuquén province of Argentina, the G6 genotype has been described in 57.1% (Kamenetzky *et al*., 2002), 36.5% (Guarnera *et al*., 2004) and 53.8% (Debiaggi *et al*., 2017) of human cysts genotyped.

Since the beginning of the study of the variability of *E*. *granulosus s.l.,* it has been suggested that species/genotypes of *E*. *granulosus s*.*l*. complex could show biological differences for example in the development of cysts. Previous studies have suggested a higher growth rate in cysts attributed to G6 compared with *E*. *granulosus s*.*s*. (Guarnera *et al*., 2004) and an apparent tropism of the G6 genotype for brain tissue (Sadjjadi *et al*., 2013). More recently, Örsten *et al*. (2020) showed that cysts from the cluster G6/7 (6 samples) were detected more frequently in non-liver locations and usually had a smaller diameter compared with 110 cysts genotyped as *E*. *granulosus s*.*s*. In the present study, we analyse differences in biological features of a large number of human cysts in CE-confirmed cases from Neuquén province caused by genotypes of *E*. *granulosus s.s.* and the G6 genotype of *E*. *granulosus s*.*l*.

## Materials and methods

### Parasite material

One hundred twenty-four human *E. granulosus* cysts were acquired from 90 CE-confirmed patients who underwent surgery at different public and private hospitals in Neuquén province in Argentina between May 2014 and July 2018. These 90 individuals represent 37.7% of all CE cases reported from Neuquén in the studied period according to the National System for Health Surveillance (Surveillance, 2019). Data about the origin, age, gender of the patient, infected organ/tissue, diameter and number of cysts per patient were recorded. Cysts from patients with hepatic CE were classified according to the WHO-IWGE classification (Brunetti *et al*., 2010). Cyst fertility was determined by detecting the presence of protoscoleces in the hydatid fluid. The viability of protoscoleces was assessed in a dye-exclusion test using 0.4% Trypan Blue and by the motility of flame cells. Protoscoleces or a piece of the germinal layer were collected from each cyst and fixed in 70% ethanol and stored at −20°C for molecular analysis, each cyst was considered a sample.

### Molecular analysis

DNA was isolated from each sample using the QIAamp DNA mini kit (QIAGEN, Germany) according to the manufacturer's instructions and stored at −20°C. To determine the cyst genotype a fragment of 366 bp of the *cox1* gene of *E. granulosus s.l.* was amplified and sequenced as previously described (Bowles *et al*., 1992; Debiaggi *et al*., 2017). The sequences were aligned and compared with reference sequences for *E*. *granulosus s.s*. G1 (accession number: U50464) and G3 (M84663), *E. equinus* G4 (M84664), *E. ortleppi* G5 (M84665) and the genotypes G6 (M84666) and G7 (M84667) and used as input in BLAST for comparison.

### Data analysis

Statistical analysis was carried out using the package InfoStat (v2017e). Univariate analysis was applied to quantitative variables (age, number of cysts per patient and cyst size), and the results were expressed as mean, one standard deviation and range. Differences in cyst size were analysed using the Student's *t*-test; Chi-square (*χ*^2^) test was used to compare categorical data. Differences were considered statistically significant when the *P* value < 0.05.

## Results

### Genetic characterization of cysts

*Echinococcus granulosus s.s.* was identified in 81/124 (65.3%) cysts available for this study; these 81 cysts were acquired from 51/90 (56.7%) patients. The G6 genotype of *E*. *granulosus s*.*l*. was identified in 43/124 (34.7%) cysts; these 43 cysts were acquired from 39/90 (43.3%) patients available for the present study (Table 1). From the 81 sequences identified as *E. granulosus s.s.*, 67 showed 100% homology with the reference sequence for the genotype G1 (U50464), 4 were identical to the reference sequence for G3 (M84663); while other 10 sequences were identical to GenBank entries previously identified in Neuquén including 8 sequences identical to G1nqnC (GU980911), 1 to G1nqnD (KC954601) and 1 to G1nqnE (JN176929). All the sequences from the 43 cysts identified as G6 shared 100% homology with the reference sequence for G6 (M84666).

**Table 1.** Distribution of genotypes of *E. granulosus sensu lato* on cysts included in this study and classification of CE patients according to the gender and age group

Genotypes	#Cysts (%)	CE patients (%)	Female (*n* = 34)			Male (*n* = 56)		
			0–18 years (%)	Adults (%)	Total female	0–18 years (%)	Adults (%)	Total male
*E*. *granulosus s*.*s*.	81 (65.3%)	51 (56.7)	6 (37.5) **	10 (62.5)	16*	14 (40.0) **	21 (60.0)	35*
G6	43 (34.7%)	39 (43.3)	0 (0)	18 (100)	18	9 (42.8)	12 (57.1)	21
Total	124	90	6	28	34	23	33	56

* *P* < 0.05.

**P < 0.005.

### Clinical characteristics of the patients: age and gender

The mean age for CE patients was 35.7 ± 22 years (range: 3–81, median = 33). There were 29/90 (32.2%) patients ≤18 years, and 61/90 (67.8%) >18 years of age out of the total of 90 CE patients included in this study (Table 2). The mean age of patients under 18 years was 11 ± 4.3 years (median = 14), and 44.1 ± 16.7 years (median = 45) for patients >18 years. Males accounted for 62.2% (56/90) of the total number of patients (*χ*^2^ = 0.1; *P* = 0.75). In the group of patients ≤18 years of age, males represented 82.8% (23/29), showing a significant difference with female cases of the same age group (*χ*^2^ = 14.23; *P* = 0.002). *E. granulosus s.s.* infected 69% (20/29) of the patients from 0 to 18 years old, while the G6 genotype was responsible for 31% (9/29) of the same age bracket showing significant differences (*χ*^2^ = 4.2; *P* < 0.05) (Table 1).

**Table 2.** Frequency of cyst location and number of cysts per patient in 90 confirmed human cystic echinococcosis patients from Neuquén, Argentina, between 2014 and 2018

Location	# Cases			# Patients	
	Any genotype (%)	*E. granulosus s.s.* (%)	G6 (%)	With 1 cyst^a^	With 2 or more cysts^a^
Liver	40 (44.4)	29 (56.9)	11 (28.2)	25	15
Lungs	38 (42.2)	15 (29.4)	23 (58.9)	35	3
Liver + peritoneum	4 (4.4)	3 (5.9)	1 (2.6)	0	4
Bone (vertebra)	4 (4.4)	3 (5.9)	1 (2.6)	1	3
Kidney	1 (1.1)	0	1 (2.6)	1	0
Pancreas	1 (1.1)	0	1 (2.6)	1	0
Retroperitoneum	1 (1.1)	1 (1.9)	0	1	0
Extra pleura	1 (1.1)	0	1 (2.6)	1	0
Total	90	51 (100)	39 (100)	65	25

a *P* < 0.05.

### Cyst localization and number of cysts per patient

The liver was the most frequently affected organ with 44.4% of the 90 CE cases, followed by the lungs with 42.2% showing a liver/lung ratio of 1.16/1 (Table 2). The rest of the *Echinococcus* cysts were found in the peritoneum, retroperitoneum, bone, kidney, pancreas and extra pleura. The number of cysts per patient ranged from 1 to 6. In total, 65/90 patients harboured only 1 cyst (72.2%) and 25 had 2 or more cysts (27.8%) (Table 2).

Of the 124 cysts analysed in this study, 63 were in the liver, from which 50 cysts were identified as *E*. *granulosus s.s.* (79.4%) and 13 as the G6 genotype (20.6%). Forty-one cysts were in the lungs, from which 17 were identified as *E*. *granulosus s.s.* (41.5%) and 24 as the G6 genotype (58.5%) (Table 3). The liver/lung ratio for *E. granulosus s.s.* was 2.13/1, while for the G6 genotype, the ratio was 0.52/1. Most of the cysts identified with the G6 genotype (30/43) were detected in extra-hepatic localization, while most of the cysts were identified as *E. granulosus s.s.* showed hepatic localization (50/81) (*χ*^2^ = 10.7; *P* = 0.001) (Table 3). Patients infected with the G6 genotype presented a maximum of 2 cysts while those infected with *E. granulosus s.s.* presented up to 6 cysts (*χ*^2^ = 11.34; *P* < 0.001).

**Table 3.** Distribution of genotypes, diameter and fertility of *E. granulosus sensu lato* cysts concerning the anatomical localization

Location	*E. granulosus sensu stricto*			G6			Total # cysts
	# Cysts	Mean diameter cm (range)	Fertility (%)	# Cysts	Mean diameter cm (range)	Fertility (%)	
Liver	50 ***	7.9 (1.8–20)	35/50 (70)	13 ***	8.3 (4.9–13)	10/13 (76.9)	63
Lungs	17 ***	8.6* (2.5–15)	12/17 (70.5)	24 ***	6.2* (3–11.6)	17/24 (70.8)	41
Bone (vertebra)	9	0.5	1/9 (11.1)	2	0.3	0/2	11
Peritoneum and Retroperitoneum	5	5.3 (3.2–8)	2/5 (40)	1	3	0/1	6
Kidney	0	–	–	1	17	1/1 (100)	1
Pancreas	0	–	–	1	–	0/1	1
Extra pleura	0	–	–	1	7.6	1/1 (100)	1
Total	81		50/81	43		29/43	124

*P < 0.05.

*** *P* = 0.001.

### Cyst size

The mean diameter of the 124 cysts was 7.0 ± 4.2 cm (range = 0.3–20 cm). For hepatic cysts, the mean diameter was 7.9 cm (1.8–20 cm), for pulmonary cysts 7.2 cm (2.5–15 cm), for vertebral bone 0.5 cm (0.3–0.5 cm) and peritoneum cysts 4.3 cm (3–8 cm). Statistical significance was found in differences in the diameter of lung cysts attributed to *E. granulosus s.s*, compared with those of the G6 from the same organ (*P* < 0.05) (Table 3).

### Fertility and viability of *Echinococcus* cysts

We observe protoscoleces in 79 out of 124 cysts (63.7%); viable protoscoleces were observed in 30.3% of those cysts. Fertile cysts were found in all locations except in the single cyst found in the pancreas. Fertility did not show significant differences between species/genotypes: 50/81 cysts attributed to *E*. *granulosus s*.*s* (61.7%) were fertile, while 29/43 cysts attributed to the G6 genotype had protoscoleces (67.4%) (*P* = 0.665) (Table 3).

### Stage of liver cysts using the WHO criteria

WHO classification was used for the ultrasound examination of 31 patients who harboured a total of 48 hepatic cysts: 20 cysts were active (CE1 and CE2), 17 were transitional cysts (CE3) and 11 were inactive cysts (CE4). *Echinococcus granulosus s.s*. was identified in cysts in the 4 stages found CE1, CE2, CE3 and CE4; the G6 genotype was characterized in cysts CE1, CE3 and CE4. In total, 84.6% (33/39) of cysts characterized as *E. granulosus s.s.* were active and transitional and 6/39 (15.3%) were inactive. In the case of G6 cysts, 55.6% (5/9) were inactive, observing significant differences with *E. granulosus s.s.* cysts (*χ*^2^ = 6.94; *P* = 0.0084).

### Geographic distribution of CE patients in Neuquén

**Fig. 1.**
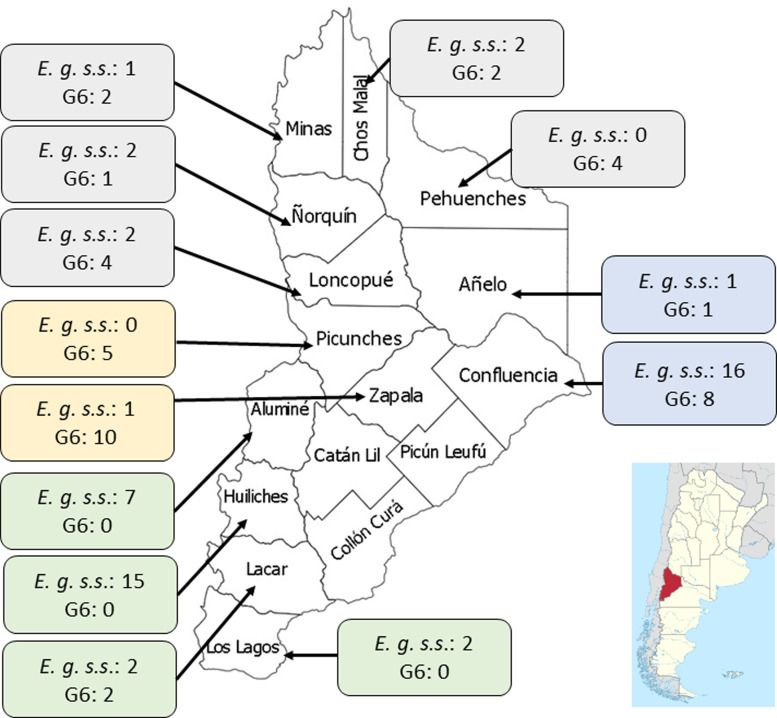
Map of the Neuquén province showing the distribution of *E. granulosus s*.*s*. (*E.g. s.s.*) and the G6 genotype identified in 90 patients from 13 districts of this province. The number of cases for each species/parasite is indicated within the boxes. District names belonging to each geographic region: (1) Northern region (grey boxes): Minas, Chos Malal, Pehuenches, Loncopue, Ñorquin; (2) Eastern region (blue boxes): Confluencia, Añelo; (3) Western region (yellow boxes): Zapala, Picunches; (4) Southern region (green boxes): Aluminé, Huiliches, Lacar, Los Lagos.

The province was divided into 4 areas (North, East, South and West) and the patients were assigned to each area according to their origin. The distribution of patients in each region was: North (20 patients), South (30), East (24) and West (16) (Fig. 1). Aggregation of human cases caused by *E*. *granulosus s*.*s*. was observed in the Southern area of Neuquén with 27/30 patients (*χ*^2^ = 18.4; *P* < 0.00001). For patients infected with genotype G6, aggregation was observed in 15/16 individuals in the Western zone (*χ*^2^ = 17.7; *P* < 0.0001) and in 13/20 patients from the Northern part of Neuquén (*χ*^2^ = 3.8; *P* < 0.05).

## Discussion

The Neuquén province is highly endemic for CE; livestock raising focused on goats is an important activity in rural areas which has favoured the presence of the G6 genotype of *E. granulosus s.l.* Furthermore, socioeconomic characteristics of the population have perpetuated the transmission of the parasite in the province despite the control programme that has been carried out since 1970. In Argentina, the mean annual incidence of human CE has been estimated to be 0.7/10^5^ inhabitants in 2018. Neuquén province has the second highest incidence of CE in the country estimated at 6.18/10^5^ (Surveillance, 2019).

Data from the present study showed that 81/124 (65.3%) of the cysts analysed from 90 patients were characterized as *E. granulosus s.s.* and 43/124 (34.7%) human cysts were identified as genotype G6. The percentage of G6 cysts found in this study is lower than the one reported previously in 2 previous studies from Neuquén in which most human cysts belonged to the G6 genotype of *E. granulosus s.l*.: 57.1% (12/21) (Kamenetzky *et al*., 2002) and 53.8% (14/26) (Debiaggi *et al*., 2017). This difference could be attributed to the fact that a greater number of cysts from almost all districts of Neuquén were province were included in this study. Nevertheless, in the current report, the percentage of *E*. *granulosus* human cysts attributed to the G6 genotype is much higher than the worldwide situation (7.34%) (Alvarez Rojas *et al*., 2014).

There are 751 853 goats in the Neuquén province which represent 17.5% of goats in the Argentinian territory (Census, 2020). Goats are raised in a transhumance model which comprises a close contact human-dog (Soriano *et al*., 2010; Debiaggi *et al*., 2017; Census, 2020). The geographical distribution of *E. granulosus s.s* and the G6 genotype in humans from the province of Neuquén was markedly heterogeneous (Fig. 1). The largest number of cysts belonging to *E. granulosus s.s* was found in patients from the South and East regions, which is characterized by the breeding of sheep and cattle and very low raising of goats (Census, 2020). The highest number of cysts belonging to the G6 genotype was found in the North and West region, which are mostly characterized by the transhumant breeding of goats.

The analysis of the distribution of CE cases according to gender and age showed that there were more male than female patients under 18 years of age, mainly in the age range between 10 and 18 years. This difference could be explained by the fact that most boys accompany their parents in herding livestock, therefore having closer contact with dogs. We found that 32.2% of the CE cases studied corresponded to children under 18 years, which suggests active transmission of the parasite exists in Neuquén despite a long-standing control programme for CE.

The most frequent anatomical localization of cysts was in the liver, followed by the lungs while some cysts were found in unusual localizations like bones (vertebra), peritoneum, kidney, pancreas, retroperitoneum and extra pleura. The total liver/lung ratio in this study was 1.16/1; this value is lower compared with the reported in a review based on 9,970 hospital records from South America, Africa, Europe, Australia and Asia (Larrieu and Frider, 2001) which showed a ratio of 2.5/1. In a previous study analysing CE patients from Neuquén, the liver/lung ratio was 4.1/1 (Pierangeli *et al*., 2007). The factors that determine the final anatomic localization of cysts are still unknown. A systematic review of the literature on human CE indicated that *E. granulosus s.s.* is located preferentially in the liver (73.4%), followed by the lungs (19.6%). The G6 genotype is reported to affect the liver (54.3%), lungs (25.7%), brain (12.9%) and other organs (7.1%) (Cucher *et al*., 2016). In this study, we observed that 78.1% of the hepatic cysts corresponded to *E. granulosus s.s.* and 21.9% corresponded to the G6 genotype, while 58.5% of the pulmonary cysts corresponded to the G6 genotype (*P* = 0.0001). Although it was observed that most G6 genotype cysts were found in the lungs, this genotype also infected other organs such as the liver, vertebrae, kidneys, peritoneum, pancreas and extra pleura. The localization of G6 genotype cysts in humans seems to be similar to that observed in goats of Neuquén and in previous studies that suggested a tropism of the G6 genotype for the lung (Bardonnet *et al*., 2002; Soriano *et al*., 2010). Sadjjadi *et al*. (Sadjjadi *et al*., 2013) also suggested an extrahepatic localization of cysts attributed to G6, in this case, the brain. However, they did not include CE patients with cysts in lungs in their study (Sadjjadi *et al*., 2013). Our results are in agreement with the recently published work by Örsten *et al*. (2020), who suggested that the cluster G6/G7 can be detected in extra-hepatic localizations more frequently than *E. granulosus s.s*. However, Örsten *et al*. (2020) compared 104 *E. granulosus s.s.* with only 6 cysts belonging to the G6/7 cluster that were not differentiated between G6 and G7. The present study shows data from a more robust number of samples including 81 cysts attributed to *E. granulosus s.s.* and 43 to the G6 genotype. Our results also suggest that cysts' localization could be related to the infecting species/genotypes of *E. granulosus s.l*. Data analysing the relationship between genetic diversity and the number of cysts per patient is scarce. The analysis of the relationship between the species/genotypes of *E. granulosus s.l.* and the number of cysts per patient showed that 42.9% of the patients infected with *E. granulosus s.s.* had more than one cyst (with a maximum of 6 cysts in 1 patient), while 9.8% of the patients infected with the G6 genotype presented up to 2 cysts (*P* < 0.001). In this study, we also found that *E. granulosus s.s.* lung cysts size was larger than lung cysts from G6 genotype (*P* < 0.05). While Guarnera *et al*. (2004) suggested that G6 genotype lung cysts were larger than *E. granulosus s.s.* lung cysts. These differences may be due to the fact that in this study we analysed more samples. However, it is important to note that cyst size depends on the time of surgery performed, which usually occurs when the patient present symptoms, while the time of infection remains unknown. These data are relevant if, for example, abdominal ultrasound screening would be used in Neuquén where several cases would be considered as false negatives since most cysts of G6 would be infecting the lungs.

No significant differences were observed in the fertility and viability of cysts between species/genotypes in this study. Few reports relate fertility to the genotype of the cyst, *E. granulosus s.s.* shows high percentages of fertility ranging between 50 and 96% (Bart *et al*., 2006; Lahmar *et al*., 2009; Omer *et al*., 2010; Macin *et al*., 2021). Patients with an indication for CE surgery usually receive pharmacological treatment with benzimidazoles to reduce the risk of secondary CE after surgery. This treatment is adjusted to the patient's characteristics (e.g., age, comorbidities) and clinical disease aspects (e.g., location and stage of the cyst, presence of complications). Thus, pharmacological treatment before surgery could affect the fertility of cysts regardless of genotype.

Despite the efforts to control parasite transmission in Neuquén since 1970 (Craig *et al*., 2017) CE remains endemic. Vaccination of livestock has not been included in control activities in Neuquén as in other parts of Argentina like Rio Negro Province (Labanchi *et al*., 2022). It remains unknown if the current EG95 vaccine would protect against the infection with the G6 genotype of *E. granulosus s.l*. (Alvarez Rojas *et al*., 2012, 2013). Considering the high prevalence of the G6 genotype in Neuquén province, it offers the perfect scenario to elucidate that question in the near future.

In conclusion, we provide strong support for the idea that *E. granulosus s.s.* presents a tropism for the liver, while the G6 genotype is more commonly found in extra-hepatic localizations (i.e., lungs). It was also observed that genotypes would influence the number of cysts per patient and the size of the lung cysts. More studies should be carried out in endemic regions worldwide to increase knowledge on clinical aspects of human CE caused by different species and genotypes of *E. granulosus sensu lato*. These facts could be considered in patient care guidelines and in CE control programs, to contribute to the improvement of the epidemiological situation of this zoonosis.

## Data Availability

There is no extra data for this paper.
